# Auranofin Releasing Antibacterial and Antibiofilm Polyurethane Intravascular Catheter Coatings

**DOI:** 10.3389/fcimb.2019.00037

**Published:** 2019-02-28

**Authors:** Hanyang Liu, Shashank Shukla, Noel Vera-González, Nagendran Tharmalingam, Eleftherios Mylonakis, Beth Burgwyn Fuchs, Anita Shukla

**Affiliations:** ^1^Center for Biomedical Engineering, School of Engineering, Institute for Molecular and Nanoscale Innovation, Brown University, Providence, RI, United States; ^2^Division of Infectious Diseases, Rhode Island Hospital, Alpert Medical School and Brown University, Providence, RI, United States

**Keywords:** antimicrobial catheter coating, antibiofilm, auranofin, polyurethane, drug delivery, *Staphylococcus aureus*, catheter-related bloodstream infection

## Abstract

Intravascular catheter related bloodstream infections (CRBSIs) are a leading cause of hospital-acquired infections worldwide, resulting not only in the burden of cost and morbidity for patients but also in the over-consumption of medical resources for hospitals and health care organizations. In this study, a novel auranofin releasing antibacterial and antibiofilm polyurethane (PU) catheter coating was developed and investigated for future use in preventing CRBSIs. Auranofin is an antirheumatic drug with recently identified antimicrobial properties. The drug carrier, PU, acts as a barrier surrounding the antibacterial agent, auranofin, to extend the drug release profile and improve its long-term antibacterial and antibiofilm efficacy and potentially the length of catheter implantation within a patient. The PU+auranofin coatings developed here were found to be highly stretchable (exhibiting ~500% percent elongation), which is important for the compliance of the material on a flexible catheter. PU+auranofin coated catheters were able to inhibit the growth of methicillin-resistant *Staphylococcus aureus* (MRSA) for 8 to 26 days depending on the specific drug concentration utilized during the dip coating process. The PU+auranofin coated catheters were also able to completely inhibit MRSA biofilm formation *in vitro*, an effect that was not observed with auranofin or PU alone. Lastly, these coatings were found to be hemocompatible with human erythrocytes and maintain liver cell viability.

## Introduction

Approximately 150 million intravascular catheters are implanted annually in the United States alone (Shah et al., 2013). Intravascular catheters are used for hemodynamic monitoring, renal replacement therapy, nutritional support, and administration of medications (Alberti et al., 2014). With the use of these intravascular devices comes a risk of catheter-related bloodstream infections (CRBSIs). Over 250,000 CRBSIs are diagnosed annually in the U.S. (Maki et al., 2006), making CRBSIs the most prevalent source of nosocomial bacteremia (Abebe et al., 2014). These infections can prolong hospital stays by ~10–20 days and increase the cost of care from $4,000 to $56,000 per patient; more importantly, CRBSIs are associated with mortality rates of 12–25% (Maki et al., 2006).

CRBSIs are predominantly caused by Gram-positive bacteria including species of *Staphylococcus aureus* (Abebe et al., 2014). Intravascular catheters can become infected by microorganisms in several ways: the catheter lumen can be contaminated prior to use, the catheter tip and cutaneous tract can be contaminated by the skin microbiome during insertion, contaminated infusate can deliver bacteria, and inserted materials can be exposed to microbes due to an existing systemic infection (Pugach et al., 1999; Abebe et al., 2014). Once bacteria are introduced to the catheter material, they can adhere and begin the process of forming a biofilm, playing a significant role in CRBSI pathogenesis (Donlan, 2002; Raad et al., 2007). Biofilms are complex, surface-attached, three-dimensional microbial colonies, consisting of bacteria embedded within a self-secreted matrix containing proteins, polysaccharides, and extracellular DNA (Donlan, 2002). Once biofilms develop on medical device surfaces they can lead to device failure (Danese, 2002) and may also spread infection by releasing planktonic cells, which can colonize downstream sites (Costerton, 1999; Stewart, 2002; Lewis et al., 2005). Eradication of biofilms is a formidable challenge due to the many sophisticated mechanisms bacteria develop to protect against host defense mechanisms and the prevalence of increased resistance against traditional antibiotic treatments (Stewart, 2002; Flemming et al., 2016; Koo et al., 2017). The biofilm matrix forms a physical barrier hindering penetration and diffusion of antimicrobial agents (Costerton, 1999; Stewart, 2002), while the low metabolic state of biofilm bacteria make them less susceptible to antibiotics (Brown et al., 1988; de la Fuente-Núñez et al., 2013). Additionally, bacteria also coordinate their physiological processes through quorum sensing (Donlan, 2002; Li and Tian, 2016), allowing the cells to communicate by releasing and responding to small molecules aiding in colonization, defense against antimicrobials, and adaptation to the microenvironment (Li and Tian, 2016). The accumulation of biofilm within the catheter can lead to the need for implant removal.

Several methods have been utilized to prevent microbial colonization of catheters. The most common methods involve the use of antimicrobial loaded or antimicrobial coated catheters. Antimicrobial agents such as cefazolin (Kamal et al., 1991), minocycline, rifampin (Raad et al., 1996), chlorhexidine, and silver sulfadiazine (Maki et al., 1997) have been deposited directly on catheter surfaces using dip coating or solvent casting methods (Darouiche et al., 1999). However, these coating strategies often lead to rapid release of the entire antimicrobial payload (Danese, 2002). In order to provide sustained drug release and long-term therapeutic efficacy, antimicrobials can be incorporated on catheters within polymeric surface coatings. Pugach et al. developed a gelatin hydrogel coating encapsulating ciprofloxacin liposomes on silicone Foley catheters, which significantly delayed bacteria colonization *in vivo* compared to uncoated catheters (Pugach et al., 1999). Fischer et al. coated polyurethane catheters with silver nanoparticles embedded in star-shaped poly(ethylene glycol)-heparin hydrogels, achieving catheter hemocompatibility and antimicrobial functionality for up to a week *in vitro* (Fischer et al., 2015). Hook et al. identified a group of polymers capable of reducing bacterial attachment up to 30-fold when compared to a commercial silver hydrogel and successfully coated catheters with these polymers demonstrating *in vivo* antibacterial efficacy (Hook et al., 2012). Fu et al. and Curtin et al. loaded bacteriophage into *Lubri-sil*®, a neutral hydrogel coating, on silicone French Foley catheters. They observed a significant reduction in viable biofilm formation by *Staphylococcus epidermidis* on the catheters over a 24 h *in vitro* exposure period (Curtin and Donlan, 2006; Fu et al., 2010). The antimicrobial efficacy of these previously reported catheter coatings has been limited to a maximum of 2 weeks.

Despite the progress that has been made, the development of drug resistance remains a significant concern while utilizing traditional antibiotic therapeutics in available catheter technologies (Danese, 2002). Thus, we sought to incorporate and examine a recently identified antimicrobial agent with therapeutic potential in a new catheter coating. Auranofin is an FDA approved antirheumatic therapeutic that is a particularly promising antimicrobial candidate, having shown antibacterial and antifungal efficacy (Cassetta et al., 2014; Harbut et al., 2015; Fuchs et al., 2016; Thangamani et al., 2016) along with potent antibiofilm efficacy (Torres et al., 2016; AbdelKhalek et al., 2018). Auranofin exhibits effective antimicrobial activity primarily against Gram-positive pathogenic bacteria including *Mycobacterium tuberculosis, Bacillus subtilis*, and *Enterococcus faecalis*, drug-sensitive and drug-resistant *Enterococcus faecium*, and *S. aureus* (Harbut et al., 2015). The minimum inhibitory concentration (MIC) of auranofin against these bacteria is as low as 0.25 μg/mL (Hassanein et al., 2017). Auranofin has a unique mechanism of action that relies on its potent inhibition of bacterial thioredoxin reductase, an important protein in thiol based redox metabolism essential in maintaining cellular processes including protection against reactive oxygen species, protein folding, and DNA synthesis (Lundstrom and Holmgren, 1990; Ritz and Beckwith, 2001; Lu and Holmgren, 2014). Inhibiting the bacterial thioredoxin reductase and disrupting the redox balance results in cell death (Bonilla et al., 2008; Debnath et al., 2012; Tejman-Yarden et al., 2013). This antibacterial drug target has been shown to highly limit the development of drug resistance (Lin et al., 2016; Sweeney et al., 2017).

Localized delivery has the potential to provide rapid antimicrobial activity, while minimizing offsite toxicity and lowering susceptibility to resistance (Brooks and Brooks, 2014). Auranofin has previously been incorporated into polymeric particles for the localized treatment of bacterial infections (Pearson et al., 2015; Díez-Martínez et al., 2016). In this work, we report the development and *in vitro* characterization of an auranofin containing polyurethane (PU) catheter coating that may have the potential to lower the incidence of CRBSIs. To the best of our knowledge, this is the first report of an auranofin containing device coating. PU is an FDA approved polymer that has been used extensively in biomedical devices for over 45 years due to its biocompatibility, mechanical flexibility (Ding et al., 2012; He et al., 2012), and low protein fouling properties (Xue and Greisler, 2003; Wilson, 2004; Maki et al., 2006). Specifically, we utilized a commercially available aromatic polyether-based PU, Texin RxT85A, to develop the auranofin encapsulating coatings reported in this work. Texin RxT85A has been used in a wide range of medical products including anesthetic connectors, flexible tubing and films, and catheters. It has also been used to fabricate drug delivery materials including nanocomposite films and nanofibers that can encapsulate and control the release of antiseptic drugs (Saha et al., 2014). Here, we demonstrate the sustained release capabilities of auranofin containing PU catheter coatings, leading to antibacterial and antibiofilm efficacy against MRSA.

## Materials and Methods

### Materials

Aromatic polyether-based PU (Texin RxT85A) was supplied by Covestro AG (Leverkusen, Germany). The antibacterial drug, auranofin, was purchased from Santa Cruz Biotechnology (Dallas, TX). All solvents, chemicals, and media, unless otherwise noted, were purchased from MilliporeSigma (St. Louis, MO). Bacto agar was obtained from BD Biosciences (San Jose, CA). Ultrapure deionized water (18.2 MΩ.cm, Milli-Q, EMD Millipore, Taunton, MA) was used in all experiments. Surflo fourteen-gauge Teflon intravenous catheters [2.15 O.D. (1.73 I.D.) × 51 mm] were supplied by Patterson Veterinary (Devens, MA). Polytetrafluoroethylene (PTFE) sheets (AMS 3651) measuring 30 cm by 30 cm with a thickness of 0.38 mm, were obtained from Amazon (Seattle, WA). Tryptic soy broth (Remel), Dulbecco's modified Eagle's medium (DMEM, Gibco), fetal bovine serum (FBS, Gibco), and precleaned microscope glass slides were purchased from Thermo Fisher Scientific (Waltham, MA). MRSA USA300 engineered to express luciferase (USA300 Lac::Lux) was supplied by Dr. Michael Hamblin at Massachusetts General Hospital (Boston, MA) (Dai et al., 2013). For cytotoxicity testing, human red blood cells (hRBCs) and human hepatoma cells (ATCC HB-8065 HepG2) were obtained from Rockland Immunochemicals (Limerick, PA) and Dr. Bryan Fuchs at Massachusetts General Hospital (Boston, MA), respectively. Cell proliferation reagent, WST-1, was obtained from Roche (Mannheim, Germany).

### Development of PU Coatings

Auranofin containing PU coatings were developed by first dissolving PU in tetrahydrofuran (THF) at a concentration of 50 mg/mL at 20°C for 24 h. Auranofin was then added to the PU solution and thoroughly mixed. This PU+auranofin mixture was then used to produce films for: (1) thickness measurement, (2) tensile testing, or (3) catheter coating for drug release and *in vitro* efficacy and cytocompatibility testing. For thickness measurements, flat PTFE substrates measuring 16 mm by 16 mm by 0.38 mm were coated via drop casting 1 mL of the PU+auranofin mixture with 0, 3, or 10 mg/mL auranofin; coatings were dried at 20°C until complete THF evaporation was noted, resulting in a dry PU+auranofin coating. For tensile testing, standalone PU+auranofin films were developed similarly to the coatings on PTFE but instead, 2 mL of the PU+auranofin mixture was drop cast onto glass slides measuring 25 mm by 75 mm. These coatings were readily peeled off of the glass and cut into rectangles measuring 12 mm by 38 mm for subsequent testing. For the catheter coatings, catheter segments measuring 10 mm in length were dipped into the PU+auranofin solution (1 catheter segment per 1 mL of PU+auranofin mixture) at auranofin concentrations of 0, 3, 10, 30, or 60 mg/mL for 24 h at 20°C. The catheters were removed from this mixture and the solvent in the coatings was allowed to evaporate at 20°C for 24 h. All coatings were stored at 4°C prior to use. Films with 0 mg/mL auranofin were denoted “PU only” coatings.

### Characterization of Coating Morphological and Mechanical Properties

The thicknesses of PU+auranofin and PU only coatings on PTFE were evaluated using a Dektak3 profilometer (Bruker, Santa Barbara, CA). Average step height was measured at three random locations on the coated material. Tensile testing of the standalone films was carried out using an Instron Series 5942 Universal Testing System (Norwood, MA) equipped with a 500 N load cell. An extension rate of 0.1 mm/s was employed until material failure was noted. The pre-yield elastic deformation region (up to 15% extension) of the engineering stress vs. strain curve was used to determine the tensile elastic modulus of the film. The interior and outer coating surfaces on the coated catheters along with non-coated catheters were imaged using a LEO Gemini 1530 scanning electron microscope (SEM, Carl Zeiss, Oberkochen, Germany). Prior to SEM imaging, the catheters were sputter coated with gold and palladium. Coated and non-coated catheters were also imaged using an inverted tissue culture trinocular microscope (AmScope, Irvine, CA) equipped with an AmScope MU500 eyepiece camera (5.1 MP Aptina Color CMOS) and 4 × objective lens.

### Quantifying Auranofin Release *in vitro*

Auranofin release from PU+auranofin coated catheters was monitored by incubating each coated catheter in 1.98 mL of tryptic soy broth supplemented with 0.25% glucose (TSBG) at 37°C with shaking at 110 rpm. Glucose supplementation of TSB has previously been shown to promote biofilm formation (Heilmann et al., 1996; Lim et al., 2004). Every 24 h, the release solutions were collected and completely replaced with fresh medium. A microdilution assay, as described previously (Shukla and Shukla, 2018), was used to determine the amount of auranofin contained in the media release samples by comparing the antibacterial activity to that of known concentrations of non-coating incorporated auranofin. Briefly, 150 μL of each TSBG release sample was transferred to 96-well plates in triplicate and serially diluted 1:1 (v/v) with TSBG. Controls of non-coating incorporated auranofin were treated similarly. MRSA USA300 (10 μL) at a final concentration of 10^5^ CFU/mL in the exponential growth phase (as determined by optical density) was added to these wells. Negative controls of media with no bacteria and positive controls of TSBG with USA300 in the absence of drug were included. Plates were incubated at 37°C with shaking at 110 rpm for ~18 h. Subsequently, the optical density (OD) of the samples was read at 600 nm using a Cytation3 microplate reader (BioTek, Winooski, VT). The normalized bacteria density was calculated using Equation (1).

(1) $$\begin{array}{l} Normalized\;bacteria\;density\\ =\frac{sample\;OD\;-\;negative\;control\;OD}{positive\;control\;OD\;-\;negative\;control\;OD} \end{array}$$

The MIC of the auranofin control against USA300 was determined as the concentration range of auranofin needed to observe a statistically significant transition of normalized bacteria density from zero to greater than zero. The amount of auranofin in the PU coating release media was then estimated by determining how many dilutions of the release media were required to reach this MIC transition point and computing a high and low estimate for the concentration of auranofin in the release solution (set by the MIC concentration range of non-coating incorporated auranofin).

Auranofin release was also monitored in water (pH 6) at 37°C for PU+auranofin coated catheters formulated with auranofin at 3 and 10 mg/mL. These coated catheters were incubated in 1 mL of water at 37°C with shaking at 110 rpm. Every 24 h, the release solutions were collected and completely replaced with fresh water. Auranofin in the water release samples was quantified using inductively coupled plasma optical emission spectroscopy (ICP-OES, Thermo Fisher, Waltham, MA). ICP-OES can detect the presence of the gold (Au) atom in the auranofin molecule. Briefly, the release samples were diluted 1:4 (v/v) with water for a total sample volume of 5 mL. This sample was injected into the ICP-OES with a radial plasma view configuration for concentrations above 1 ppm of auranofin and with an axial plasma view configuration for concentrations below 1 ppm. The concentration of auranofin in the release solutions was calculated by comparing the intensity of the signal obtained at the characteristic wavelength range of Au (242.79–242.80 nm) against that of a known auranofin standard examined concurrently.

### Assessing Coating Antibacterial and Antibiofilm Efficacy *in vitro*

Antibacterial and antibiofilm activity of PU+auranofin catheter coatings was examined against USA300, a community-associated MRSA strain. Kirby-Bauer and broth bacteria survival assays on the coated catheters and on their release solutions were conducted, respectively. For the Kirby-Bauer assay, USA300 in its exponential growth phase at a concentration of 10^8^ CFU/mL was spread on tryptic soy agar plates. PU+auranofin coated and uncoated catheters were cut in half lengthwise using a scalpel. Both the inner and outer surfaces of the catheters were placed in direct contact with the bacteria seeded agar and incubated for 24 h at 37°C. These plates were then photographed using a Canon PowerShot S110 digital camera (Tokyo, Japan).

Long-term antibacterial activity of the coated catheters was confirmed using MRSA microdilution assays as described in the section Quantifying Auranofin Release *in vitro*. The efficacy of these coatings against a greater MRSA challenge than a standard microdilution assay (2 x 10^6^ CFU/mL vs. 10^5^ CFU/mL, respectively) was investigated. Coated catheters were incubated in 1.98 mL of TSBG medium with shaking at 110 rpm at 37°C. The release medium was collected every 24 h and replaced with fresh TSBG medium. A 20 μL suspension of USA300 was added to the release solution to obtain a final bacteria concentration of 2 × 10^6^ CFU/mL. This bacterial suspension was incubated with shaking at 110 rpm at 37°C for 24 h. The OD of the samples was measured at 600 nm using a Thermo Scientific Spectronic 2000 Visible Spectrophotometer (Waltham, MA). Negative controls of media with no bacteria and positive controls of TSBG with USA300 in the absence of drug were included. A normalized bacteria density was computed using Equation (1) for all test samples. Additional release samples were tested for bacterial growth inhibition until no inhibition of bacterial growth was observed. An identical assay was conducted to examine the effect of coating time on antibacterial activity over time. For this study, coatings were formulated as described in section Development of PU Coatings, except that coating times were varied (5 s, 1 h, 1 day, or 7 days).

The antibiofilm activity of PU+auranofin coatings was also examined. PU+auranofin coatings with 3 or 10 mg/mL auranofin utilized in the coating process, along with PU only coatings and variations of these coatings, in which vancomycin replaced auranofin, or only auranofin (3 mg/mL) was used without any PU, were examined in these studies. Vancomycin coated catheters were first dip coated in an ethanol solution containing the drug at either 3 or 10 mg/mL for 24 h at 20°C. These catheters were then removed from the ethanol solution and allowed to dry for 24 h at 20°C. The catheters were subsequently dip coated in 50 mg/mL PU in THF for 24 h at 20°C, followed by a complete drying of these coatings at 20°C. Coated or uncoated catheter segments were placed in 1 mL USA300 bacterial suspensions at a concentration of 10^4^ CFU/mL in TSBG at 37°C with shaking at 110 rpm for 2 h. The samples were then removed from this suspension and rinsed 3 times with fresh TSBG to remove any unattached bacteria. The rinsed catheter segments were placed in new sterile vials containing 5 mL of fresh TSBG every 12 h. After 2 days, the bacterial burden on the catheters was evaluated by examining the level of bacterial bioluminescence on the catheters using an *in vivo* imaging system (IVIS Lumina III, PerkinElmer, Waltham, MA). Following IVIS imaging, the biofilms were disrupted by placing the catheter segments in 5 mL of 1 × phosphate buffered saline (PBS) and subjected to sonication at ~40 kHz for 7 min (Fisher Scientific FS30) followed by vortexing for 1 min. The samples were serially diluted in TSBG, plated on tryptic soy agar, and the colony forming units (CFUs) were counted.

### Examining Coating Biocompatibility *in vitro*

The biocompatibility of PU+auranofin coatings and controls was evaluated by examining erythrocyte lysis and human hepatoma cell (ATCC HB-8065 HepG2) viability upon exposure to coated catheters or catheter incubated media, respectively. Hemolysis was examined as previously described (Gwisai et al., 2017; Zhou et al., 2017) by incubating hRBCs with PU+auranofin coated catheters (3, 10, 30, and 60 mg/mL auranofin coating concentration), PU only coatings, auranofin only coatings (3 mg/mL coating concentration), and uncoated catheters. Catheters were incubated with 1 mL of 2% (v/v) hRBCs in 24-well plates for 1 h at 37°C. Negative controls containing no catheter and only hRBCs were also included. Positive controls of the 2% hRBCs suspension incubated with 0.1% v/v Triton X-100 were included. After incubation, the plates were centrifuged at 500 × g for 5 min. A 50 μL aliquot of the supernatant from each well was transferred to a 96-well plate. Absorbance at 540 nm was quantified using a SpectraMax M2 plate reader (Molecular Devices, San Jose, CA) to measure hemolysis. Normalized hemolysis was calculated using Equation (2).

(2) $$\begin{array}{l} \;Normalized\;hemolysis\\ =\frac{sample\;abs\;-\;negative\;control\;abs}{positive\;control\;abs\;-\;negative\;control\;abs} \end{array}$$

The viability of HepG2 cells exposed to PU+auranofin coating release solutions was assessed using a colorimetric assay with WST-1. HepG2 cells were maintained in DMEM supplemented with 10% FBS at 37°C with 5% CO_2_. PU+auranofin coated catheters (3 mg/mL auranofin coating concentration), PU only coatings, and uncoated catheters were incubated in HepG2 culture media for 24 h at 37°C. HepG2 cells were seeded at a density of 3.125 × 10^6^ cells/cm^2^ in polystyrene tissue-culture treated 96-well plates (Corning, Corning, NY) and immediately incubated with 100 μL of the different catheter incubation medias at 37°C with 5% CO_2_. Non-coating incorporated auranofin was also included at concentrations of 0.5–32 μg/mL. After 20 h, 10 μL of WST-1 was added to each well and the plates were incubated for 4 h at 37°C with 5% CO_2_. The absorbance (abs) of each well was measured at 450 nm using a SpectraMax M2 UV-Vis microplate reader (Molecular Devices, San Jose, CA). Normalized cell viability was calculated using Equation (3).

(3) $$\begin{array}{l} Normalized\;cell\;viability\\ =\frac{sample\;abs\;-\;negative\;control\;abs}{positive\;control\;abs\;-\;negative\;control\;abs} \end{array}$$

### Statistical Analysis

All experiments were conducted in triplicate at minimum. All data is reported as mean ± standard deviation. Statistical significance was calculated using GraphPad Prism with either a two-tailed *t*-test or one- or two-way analysis of variance (ANOVA) with Tukey's *post-hoc* analysis, as appropriate. Data was considered statistically significant for *p* < 0.05.

## Results and discussion

### *In vitro* Auranofin Release From Coated Catheters and Antibacterial Efficacy

In this work, PU+auranofin catheter coatings were developed to combat complications such as bacteria attachment, infection, and biofilm development that can occur with extended catheter use (Trautner and Darouiche, 2004). To the best of our knowledge, auranofin has not previously been used in device coatings. We sought to determine if auranofin was released from PU coatings at concentrations effective against planktonic MRSA. Initial investigations were performed with PU+auranofin coated catheters formulated from four concentrations of auranofin coating solution (3, 10, 30, and 60 mg/mL). Auranofin, unlike many other antimicrobial agents such as vancomycin, which has a measurable absorbance (Shukla and Shukla, 2018), is not readily detectable via absorbance or fluorescence spectroscopy without modification. Therefore, MRSA growth inhibition in a microdilution assay was utilized to estimate the concentrations of auranofin present in coated catheter release solutions. In this technique, serial dilutions of the release samples are made and incubated with MRSA. The most diluted solution able to inhibit bacterial growth is considered the upper range of the MIC of the drug in the release sample. By multiplying the dilution factor used to reach this concentration with the measured MIC of the drug, a concentration of drug in the release solution can be estimated.

**Figure 1:**
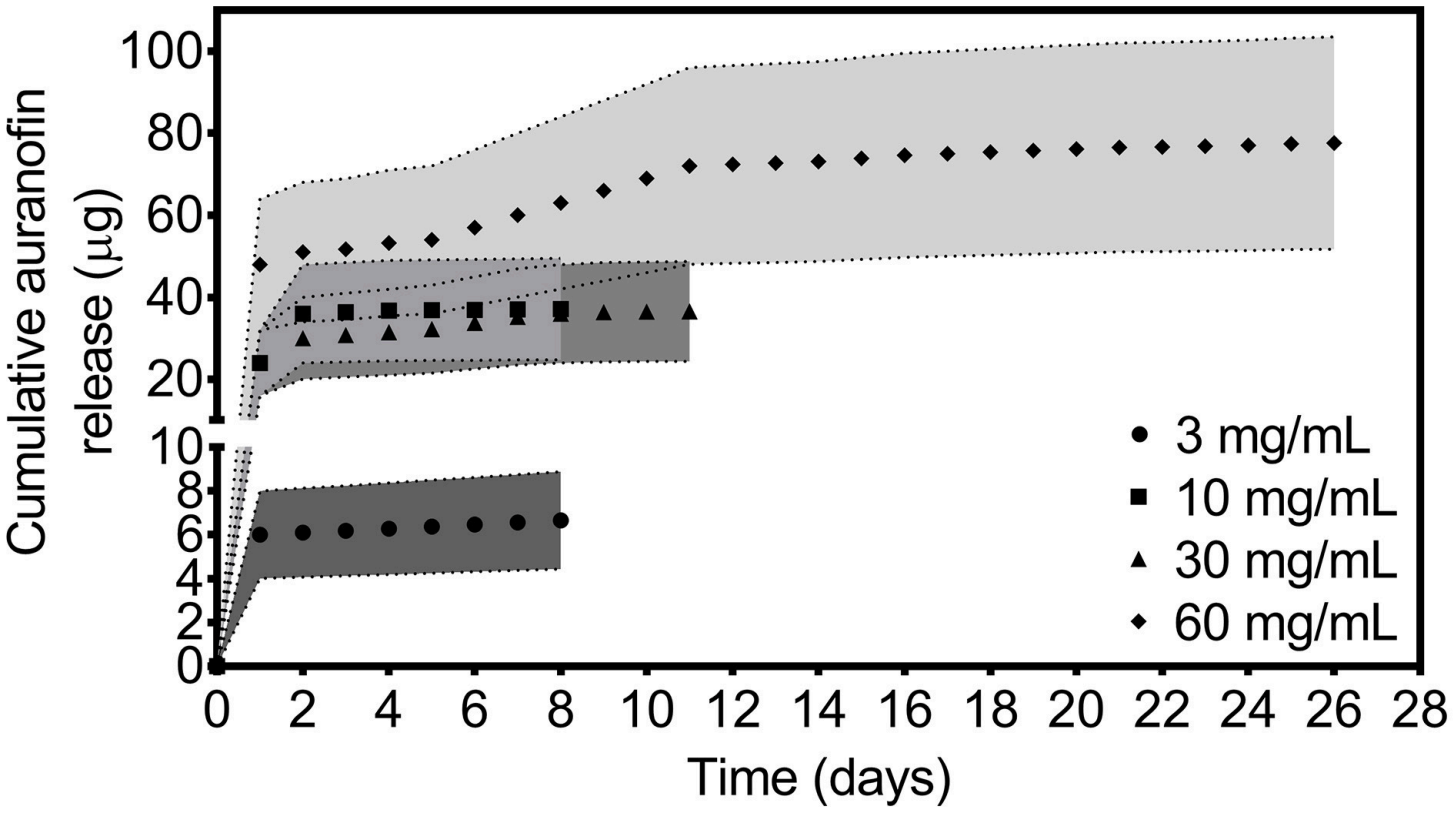
*In vitro* release profile of auranofin in TSBG medium for PU+auranofin coated catheters formulated at 4 different auranofin coating concentrations (3, 10, 30, and 60 mg/mL). Release was evaluated by examining MRSA USA300 inhibition. Data is represented as a range set by the upper and lower limits of the auranofin MIC for each day at which the release solution auranofin concentration was above MIC for USA300 (*n* = 3).

Figure S1 shows normalized MRSA density over a range of concentrations for non-coating incorporated auranofin. The MIC of non-coating incorporated auranofin against USA300 was determined to be 0.063 μg/mL, consistent with previous reports (Harbut et al., 2015; Fuchs et al., 2016; Thangamani et al., 2016). We observed a transition of MRSA growth to no growth between auranofin concentrations of 0.031 and 0.063 μg/mL. Using this concentration range for non-coating incorporated auranofin and assuming no change in auranofin activity caused by the coating process, the auranofin release profile from the PU+auranofin catheter coatings was determined. Figure 1 shows the release profile of all PU+auranofin coatings tested as a range estimated by the dilution factor required to reach MIC values. Catheters with coatings formulated using 3 and 10 mg/mL auranofin solutions exhibited effective drug release above MIC values over a period of 8 days. Raising the drug concentration in the coatings during formulation to 30 or 60 mg/mL extended auranofin release from 8 days to 11 and 26 days, respectively. A large auranofin release was observed for all coating formulations in the first 24 h followed by a slow extended release. As shown in Figure 1, for the 3 mg/mL auranofin coating concentration, ~90% of the total auranofin eluted by 8 days was released in the first day. Interestingly, 10, 30, and 60 mg/mL PU+auranofin samples all had similar percentages of auranofin released in the first day (~65, 66, and 62% of the total auranofin released by 8, 11, and 26 days, respectively). The similar burst release for the higher auranofin coating concentrations suggests that at these concentrations, the burst release of auranofin is independent of the amount of auranofin loaded on the catheters. The cumulative release of auranofin was increased from ~7 μg for a 3 mg/mL auranofin coating concentration to ~37 μg for a 10 and 30 mg/mL auranofin coating concentration and 78 μg for a 60 mg/mL auranofin coating concentration. Given that the bacterial MIC is reached with the lowest auranofin formulation concentration, it is perceivable that this formulation could be effective in inhibiting bacterial accumulation on the implant material.

It is possible that the final auranofin release values do not represent the total auranofin loading in a single 10 mm catheter segment and that some auranofin may still release below MIC values which are not readily detectable. ICP-OES, which is capable of detecting the gold atom in auranofin molecules in non-complex solvents (e.g., water rather than PBS or media), was utilized to evaluate auranofin release in deionized water (pH 6) at 37°C from PU+auranofin catheters formulated using 3 and 10 mg/mL auranofin solutions. At 8 days, 95 ± 31 μg of auranofin had released from the 3 mg/mL auranofin coatings and 319 ± 62 μg of auranofin was released from the 10 mg/mL coatings. As with the media release studies quantified using bacterial microdilution methods, a greater cumulative release was observed from the PU+auranofin coatings formulated using 10 mg/mL auranofin compared to 3 mg/mL auranofin at 8 days. However, the values quantified for water release were significantly greater than those observed in media. These differences may arise from the differences in the release environment for the two methods used. Media components may adsorb onto or absorb into the catheter coating over time and potentially form interactions with auranofin or PU, slowing drug release. The water environment lacks these interactions and may therefore enable a greater release. The differences in pH (pH 7.4 for the media vs. 6 for the water) may also factor in, as has previously been observed for polyurethane materials (Chen et al., 2014).

**Figure 2:**
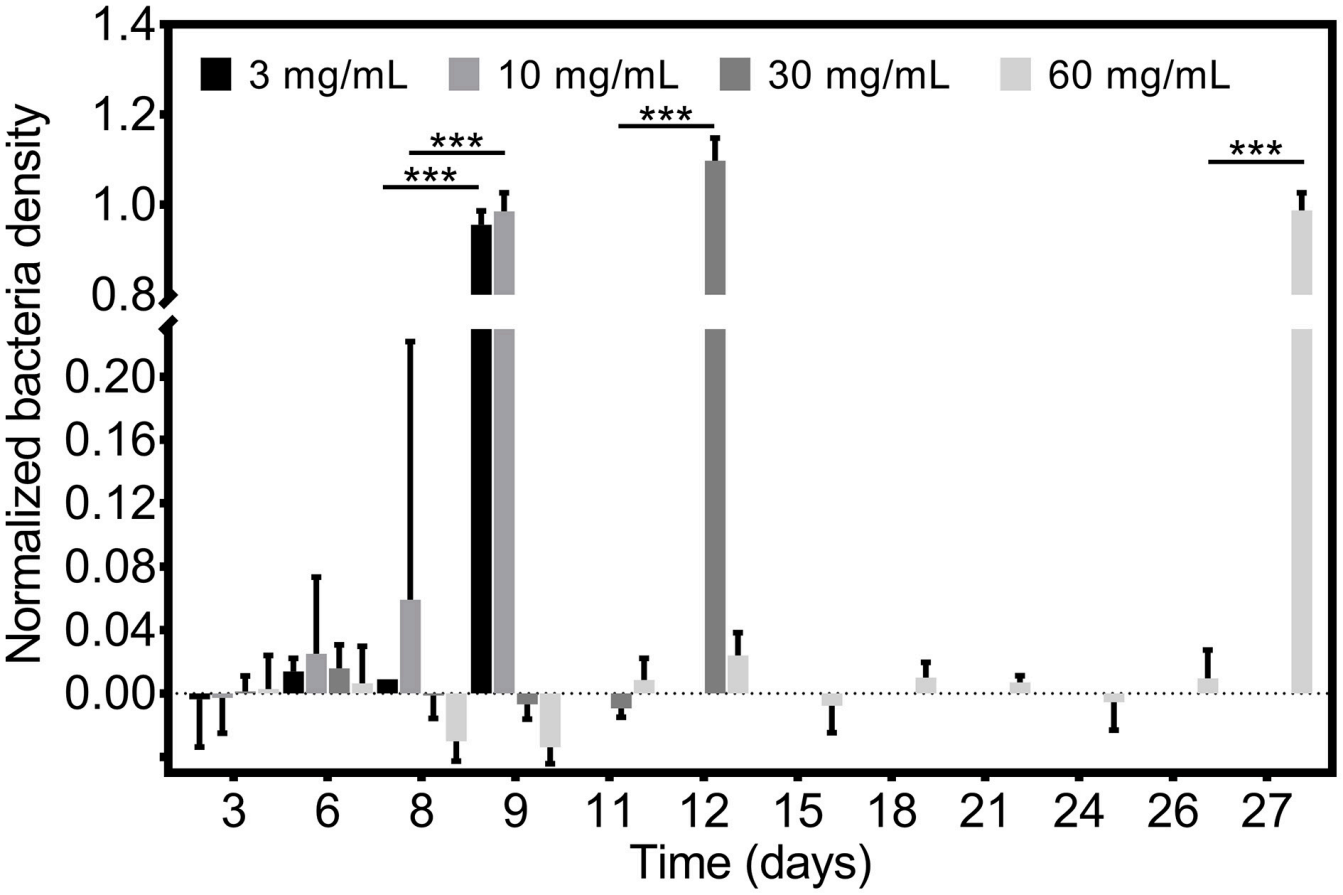
Antibacterial efficacy of PU+auranofin coated catheters. PU+auranofin coatings formulated at 4 different auranofin coating concentrations (3, 10, 30, and 60 mg/mL) were incubated with 2 × 10^6^ CFU/mL MRSA USA300 and normalized bacteria density was determined following 18 h. Data are shown as mean ± standard deviation where ***p < 0.001 indicates significance between days using two-way ANOVA with Tukey's *post-hoc* analysis (*n* = 3).

The *in vitro* auranofin release profile studies confirmed planktonic bacterial inhibition by individual catheter release samples at PU+auranofin coating compositions formulated using 3, 10, 30, and 60 mg/mL auranofin. Given the potent activity of auranofin at low concentrations, we examined whether auranofin release from these coatings was also able to inhibit the growth of MRSA at a 20 times greater bacterial concentration than the standard microdilution assay. Figure 2 shows the efficacy of MRSA growth inhibition for this greater bacterial challenge (2 × 10^6^ CFU/mL) upon bacterial incubation with PU+auranofin coating release solutions collected over time. We found that the coating release samples were able to completely inhibit bacterial growth for samples collected at time points identical to the microdilution assays conducted at lower bacteria concentrations. Namely, PU+auranofin coatings formulated with the 3 and 10 mg/mL auranofin were effective over 8 days, while those formulated with 30 and 60 mg/mL auranofin were effective for 11 and 26 days, respectively.

**Figure 3:**
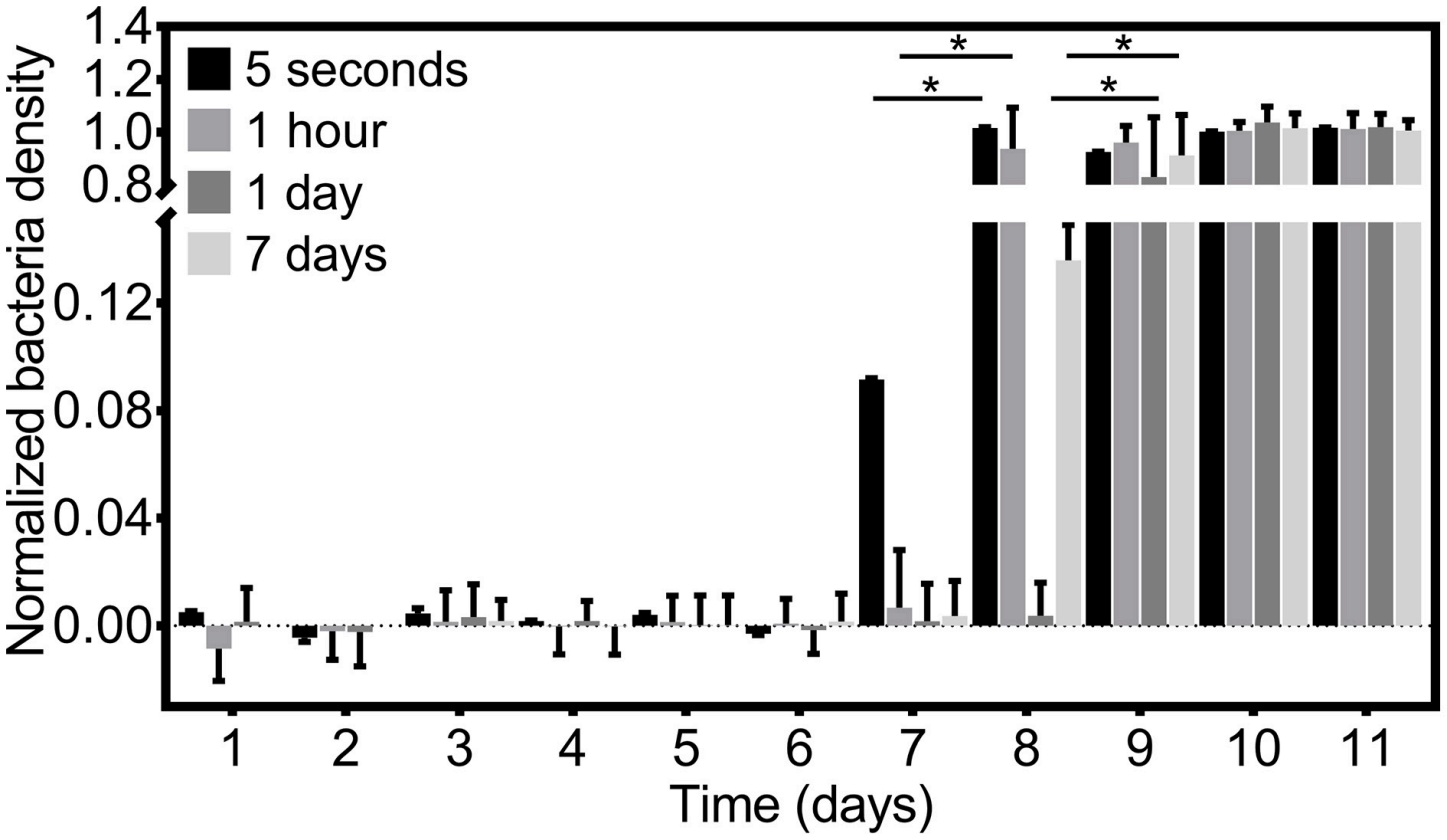
Antibacterial efficacy of PU+auranofin coated catheters formulated by varying coating times. PU+auranofin coatings formulated at 4 different coating times (5 s, 1 h, 1 day, and 7 days) at an auranofin coating concentration of 3 mg/mL were incubated with 2 × 10^6^ CFU/mL MRSA USA300 and normalized bacteria density was determined following 18 h. Data are shown as mean ± standard deviation where *p < 0.05 indicates significance between days using two-way ANOVA with Tukey's *post-hoc* analysis (*n* = 3).

Having confirmed multi-day *in vitro* efficacy of all coating formulations examined, we investigated whether changing the coating formulation process could affect efficacy. Specifically, we determined whether the catheter coating time in the PU+auranofin solution changed its efficacy. Holding the auranofin concentration in the coating solution constant at 3 mg/mL, we examined coating times of 5 s, 1 h, and 7 days in addition to the 1 day coating time utilized for all other experiments. Release samples taken over time from each of these coatings were examined for their bacterial growth inhibition efficacy using a 2 × 10^6^ CFU/mL MRSA concentration as shown in Figure 3. We observed that catheters coated for 1 and 7 days behaved similarly, inhibiting MRSA growth over 8 days. Interestingly, catheters coated for 5 s and 1 h exhibited antibacterial activity against MRSA for 7 days. These findings suggested that even short coating periods lead to significant drug incorporation on the catheter capable of releasing and inhibiting bacterial growth over a period of 1 week. Effective above MIC release can be extended by 1 day if the coating duration is increased. For future translation of these materials, rapid production is possible without significantly compromising efficacy.

**Figure 4:**
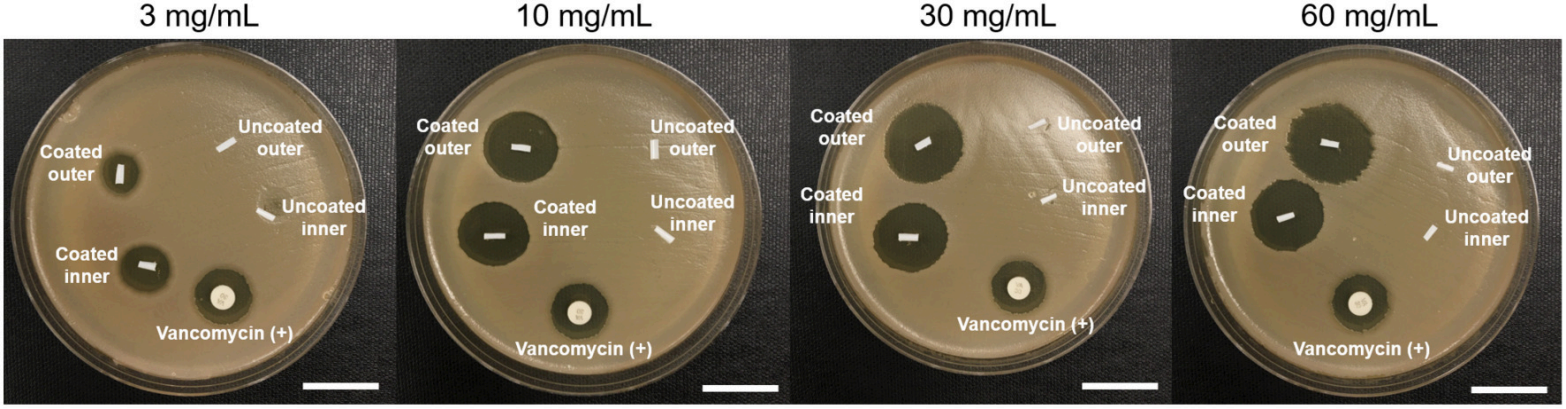
Examining the effect of PU+auranofin catheter inner and outer surfaces on MRSA USA300. Tryptic soy broth agar coated with USA300 was exposed to the inner and outer surfaces of coated bisected catheters for PU only and PU+auranofin coated materials formulated at 4 different auranofin concentrations (3, 10, 30, and 60 mg/mL). Vancomycin discs containing 30 μg of vancomycin were used as a positive control (Scale bar = 10 mm).

Next we examined whether PU+auranofin coated catheters were able to inhibit bacterial growth using both the inner and outer surface of the coated catheters. The results of a Kirby Bauer assay using PU+auranofin coated catheters formulated using 3, 10, 30, and 60 mg/mL auranofin are shown in Figure 4. For these experiments coated catheters were cut in half lengthwise and plated with either the inner surface of the catheter (i.e., the catheter lumen) or the outer surface face down on MRSA coated agar; controls of uncoated catheters were also included. A clear zone of inhibition surrounded all PU+auranofin coated catheter samples regardless of whether the inner or outer surface was exposed to the bacteria. In contrast, the uncoated samples did not exhibit any bacterial growth inhibition. Positive controls of 30 μg vancomycin discs were included and performed as expected with an average zone of inhibition diameter of 1.48 ± 0.2 cm. Quantitative comparison between inner and outer surface catheter coatings in terms of drug loading and efficacy are difficult to make as small differences in sample size and shape can alter the shape and size of the zone of inhibition that is observed surrounding these samples. Further, a degree of dose dependent release was suggested by the smaller clearing generated in the presence of the catheter material coated with 3 mg/mL auranofin compared to the other coating formulations examined.

Overall, our investigations demonstrated that catheter coatings generated with 3 and 10 mg/mL auranofin were highly effective in inhibiting bacterial growth albeit over shorter timescales compared to the 30 and 60 mg/mL auranofin coating conditions. For the purposes of *in vitro* characterization, we proceeded with using the 3 and 10 mg/mL auranofin coating concentrations for further analysis of coating morphology, mechanical properties, antibiofilm efficacy, and cytocompatibility. For conditions that may require lengthier application, the greater auranofin coating concentrations of 30 and 60 mg/mL are possible options that can be explored.

### Catheter Coating Morphology and Mechanical Properties

**Figure 5:**
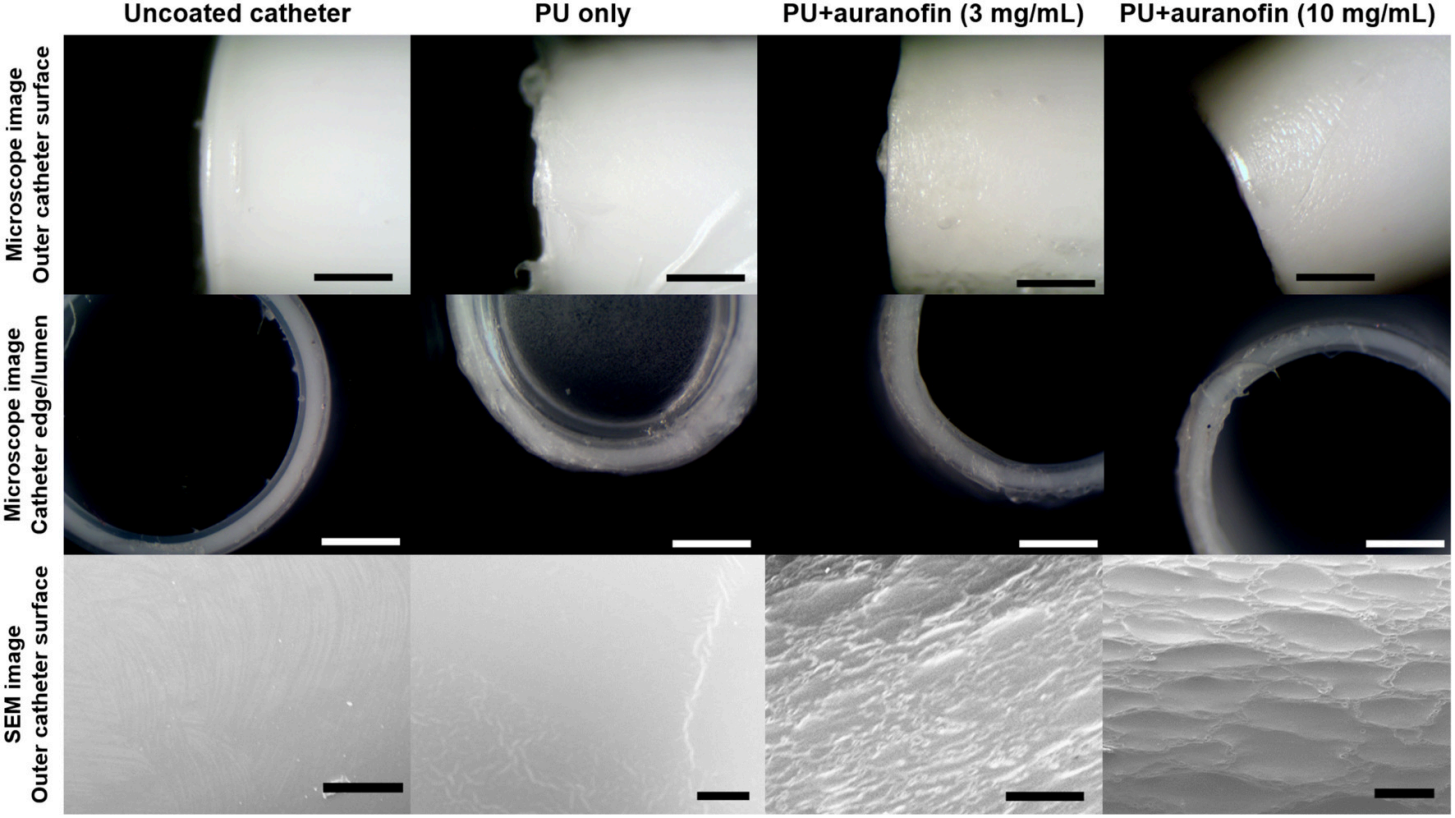
Catheter coating morphology examined via light microscopy and scanning electron microscopy. Coatings formulated with PU only and PU+auranofin at auranofin concentrations of 3 and 10 mg/mL coated for 1 day are shown [Scale bars = 500 μm (light microscope) and 50 μm (SEM)].

Having determined that PU+auranofin coatings lead to effective auranofin release and MRSA inhibition, we sought to determine if coating the catheter altered the implant material. Light microscope images of the coatings formulated with PU+auranofin solutions containing 3 and 10 mg/mL auranofin demonstrate a slightly rough exterior, with discernible differences from the PU only coated Teflon catheter (Figure 5). SEM imaging of the catheters confirmed surface texturization in the PU+auranofin coated catheters compared to the PU only coated catheters (Figure 5). Compared with PU only coatings, the added surface texture may result from the interaction of auranofin with the PU during the drying process, preventing a completely smooth surface from forming.

**Figure 6:**
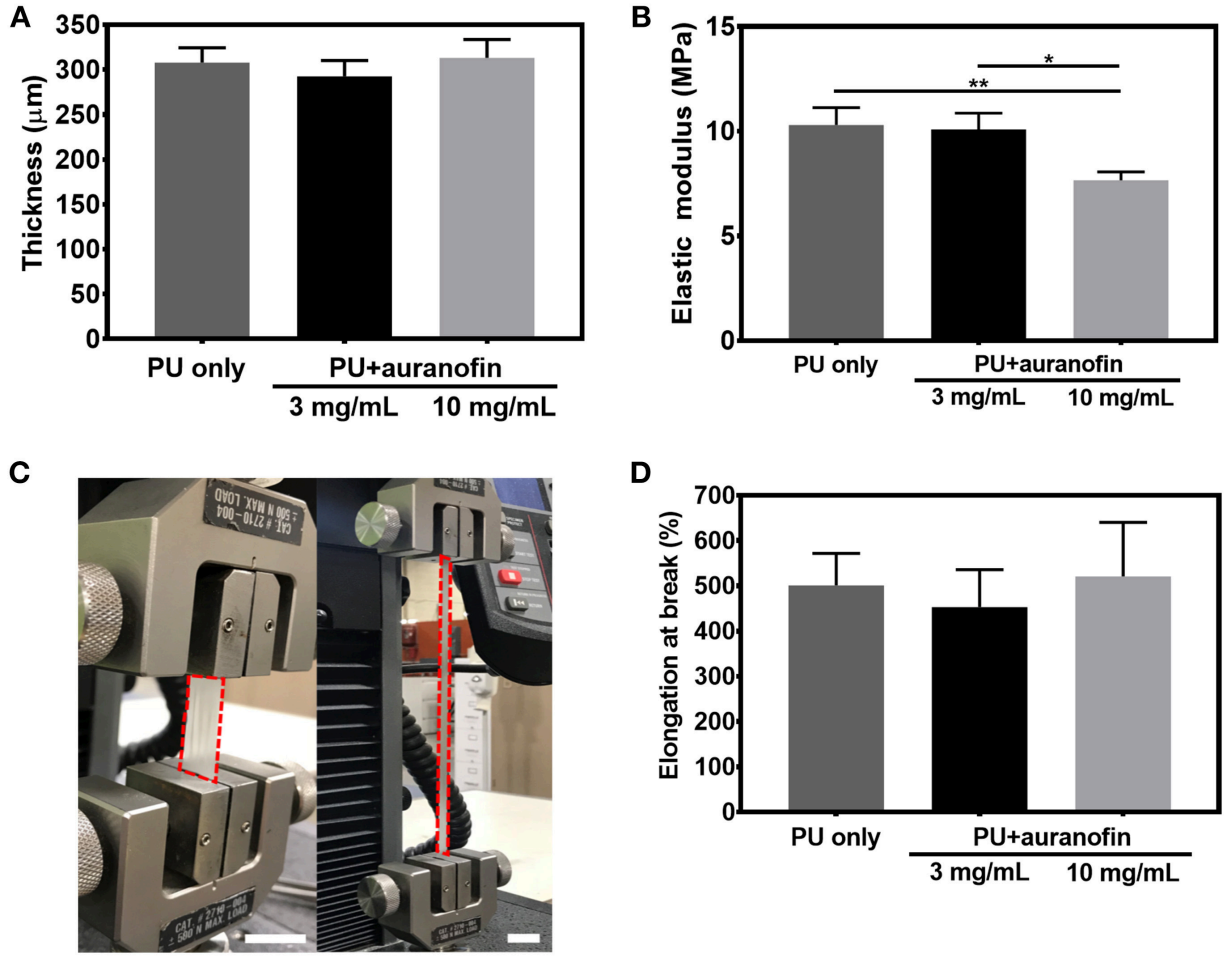
Thickness and mechanical properties of coated catheters. **(A)** Thickness of PU only and PU+auranofin (3 and 10 mg/mL auranofin coating concentration) on PTFE. **(B)** Tensile elastic moduli of PU and PU+auranofin (3 and 10 mg/mL auranofin coating concentration) standalone coatings. **(C)** Representative digital image displaying elongation of standalone PU+auranofin coating formulated with 3 mg/mL auranofin during tensile testing nearing failure (scale bars = 25 mm). **(D)** Percent elongation at break of PU and PU+auranofin (3 and 10 mg/mL auranofin coating concentration) standalone coatings. Data are shown as mean ± standard deviation with *p < 0.05 and **p < 0.001 for moduli values between samples analyzed using one-way ANOVA with Tukey's *post-hoc* analysis (*n* = 3).

The thickness of these coatings on flat PTFE sheets was examined via profilometry for PU+auranofin coatings formulated with auranofin coating concentrations of 3 and 10 mg/mL. Figure 6A shows the average thickness of PU only and PU+auranofin coatings. The average thickness of PU only coatings was 307.7 ± 16.6 μm; auranofin loaded PU coatings had average thicknesses of 292.5 ± 17.7 and 313.1 ± 20.5 μm for 3 and 10 mg/mL auranofin, respectively. The presence of auranofin did not lead to statistically significant changes in coating thicknesses between these three groups, despite the effect on coating morphology.

To further evaluate the mechanical properties of the PU+auranofin coatings, tensile tests were performed on standalone coatings. As seen in Figure 6B, PU coating stiffness decreased when 10 mg/mL but not 3 mg/mL auranofin was included in the coating process compared to PU only. PU only coatings exhibited an elastic modulus of 10.3 ± 0.8 MPa vs. 10.1 ± 0.8 MPa and 7.7 ± 0.4 MPa for PU+auranofin coatings formulated from 3 to 10 mg/mL auranofin, respectively. The decreased stiffness may result from disruption of the hydrogen bonding in the PU hard segments, which is known to reinforce the material (Shoeib et al., 2010). This effect has been observed with poly(ethylene glycol), where the oxygen atoms in the backbone act as hydrogen bond acceptors that weaken the PU hard segments (Park et al., 2001). The auranofin molecule has nine hydrogen bond acceptors and may have a similar effect. The coatings are highly stretchable as seen in Figure 6C. There was no statistical difference between the percent elongation at break (~500%) between PU coatings formulated with and without auranofin (Figure 6D), in agreement with what has previously been reported for PU coatings (La Francesca et al., 2018). Overall, the incorporation of auranofin does not appreciably impact the tensile properties of these coatings compared to PU only, maintaining the high degree of stretchability which will be important for future clinical use on catheters.

### *In vitro* Antibiofilm Efficacy of Coated Catheters

Having examined the morphological and mechanical properties of the PU+auranofin coatings, we next determined how biofilm formation was affected by the drug coatings. Auranofin was previously suggested to inhibit preformed *S. aureus* biofilms within 2 h of exposure, although with limited bactericidal activity likely due to the lack of metabolic activity required for target effectiveness within biofilm bacteria (Torres et al., 2016). Our work expands upon these previous findings, now examining the effect that auranofin has on biofilm prevention, as would be the case for a newly introduced medical implant.

**Figure 7:**
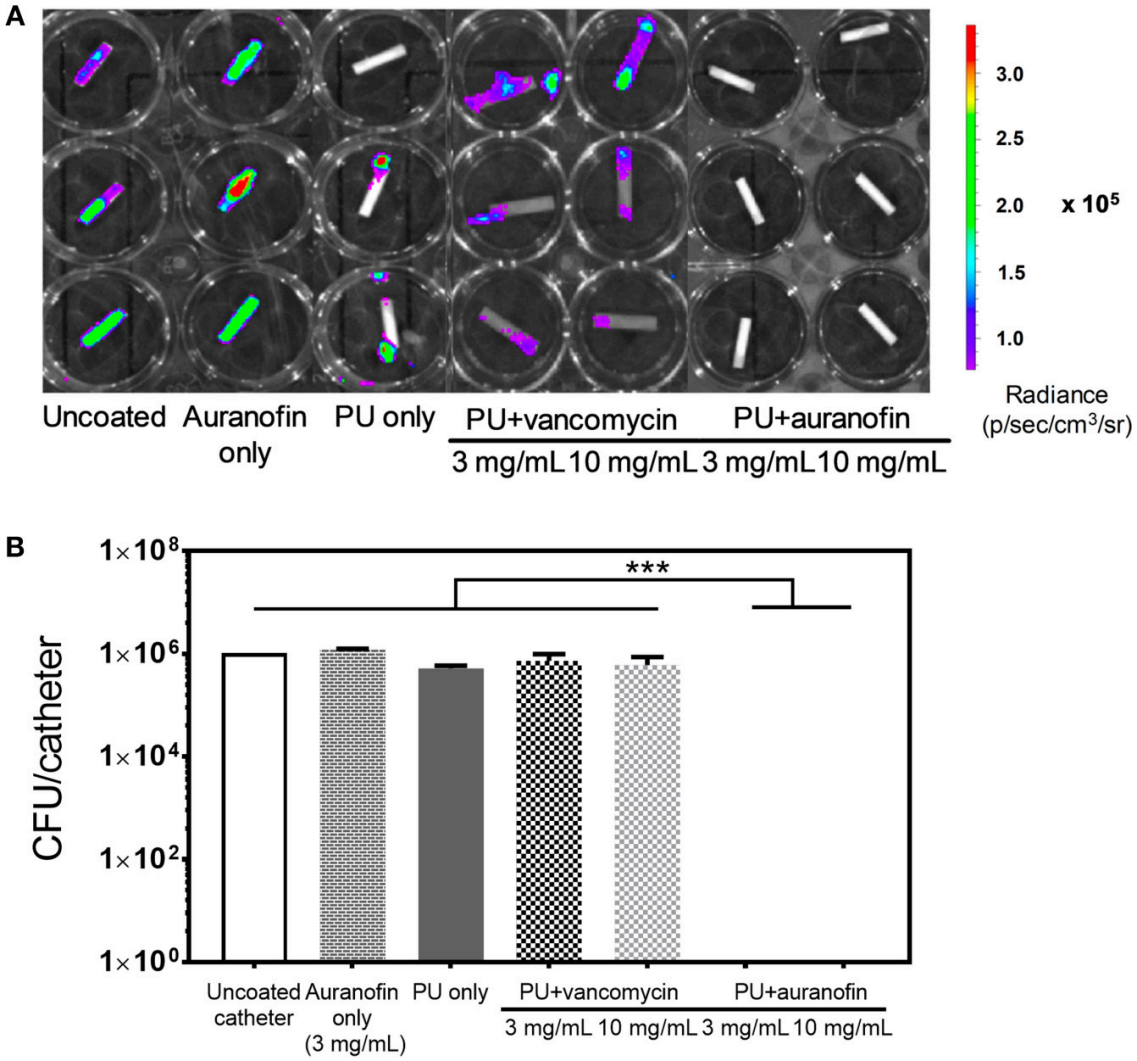
Antibiofilm efficacy of coated catheters. **(A)** Bioluminescence (radiance) of MRSA USA300 Lac::Lux examined using IVIS. Catheters were either uncoated or coated with auranofin only (at a 3 mg/mL coating concentration), PU only, PU+vancomycin, or PU+auranofin at 2 different drug coating concentrations (3 and 10 mg/mL). Samples were exposed to USA300, rinsed, and potentially attached biofilms were allowed to mature over 2 days prior to measurement. **(B)** The colony forming units of USA300 recovered from the different catheter groups imaged in **(A)**. Data are shown as mean ± standard deviation where ***p < 0.001 indicates significance between CFU for the samples evaluated using one-way ANOVA (*n* = 3).

To further examine the potential for using PU+auranofin coated catheters clinically, coated catheter segments formulated using 3 and 10 mg/mL auranofin were exposed to MRSA and then examined for biofilm formation over 48 h. PU+vancomycin, auranofin only (lacking PU, formulated with 3 mg/mL auranofin), and PU only coatings were also examined along with uncoated catheters. Vancomycin, a potent glycopeptide antibiotic highly effective against MRSA (Abebe et al., 2014), was loaded onto the catheters as a control to test its antibiofilm efficacy in comparison to auranofin coatings. Biofilm accumulation on catheter segments was visualized using an IVIS imaging system as seen in Figure 7A with luminescence indicating the presence of bacteria. Subsequently, the number of colony forming units (CFUs) attached on catheters was quantified by detaching the colonies and counting, as shown in Figure 7B. With the exception of the PU+auranofin coatings, all catheters tested exhibited bacterial luminescence. Both PU+auranofin formulations completely inhibited bacterial attachment.

We saw that PU+vancomycin and auranofin only coatings did not exhibit any statistical difference in bacterial CFU attachment as compared to uncoated catheters. Due to vancomycin hydrophilicity, we hypothesize that vancomycin is released rapidly from these coatings, leading to a lack in efficacy in preventing biofilm formation. Auranofin only coatings are also likely highly unstable due to the lack of a polymer carrier and are similarly unable to prevent bacterial attachment.

Interestingly, PU only coatings showed a 1-log reduction in bacterial attachment as compared to uncoated catheters. This antibiofilm activity of PU has previously been observed (Martínez-Martínez et al., 1990; Lopez-Lopez et al., 1991; Zdrahala and Zdrahala, 1999) and is likely due to the smooth PU surface as we observed with SEM. Overall, formulating auranofin in a PU catheter coating enabled both bacterial growth inhibition in planktonic cultures over time and complete prevention of bacterial surface attachment, which was not possible for either PU or auranofin alone.

### *In vitro* Cytotoxicity Evaluation of Coated Catheters

**Figure 8:**
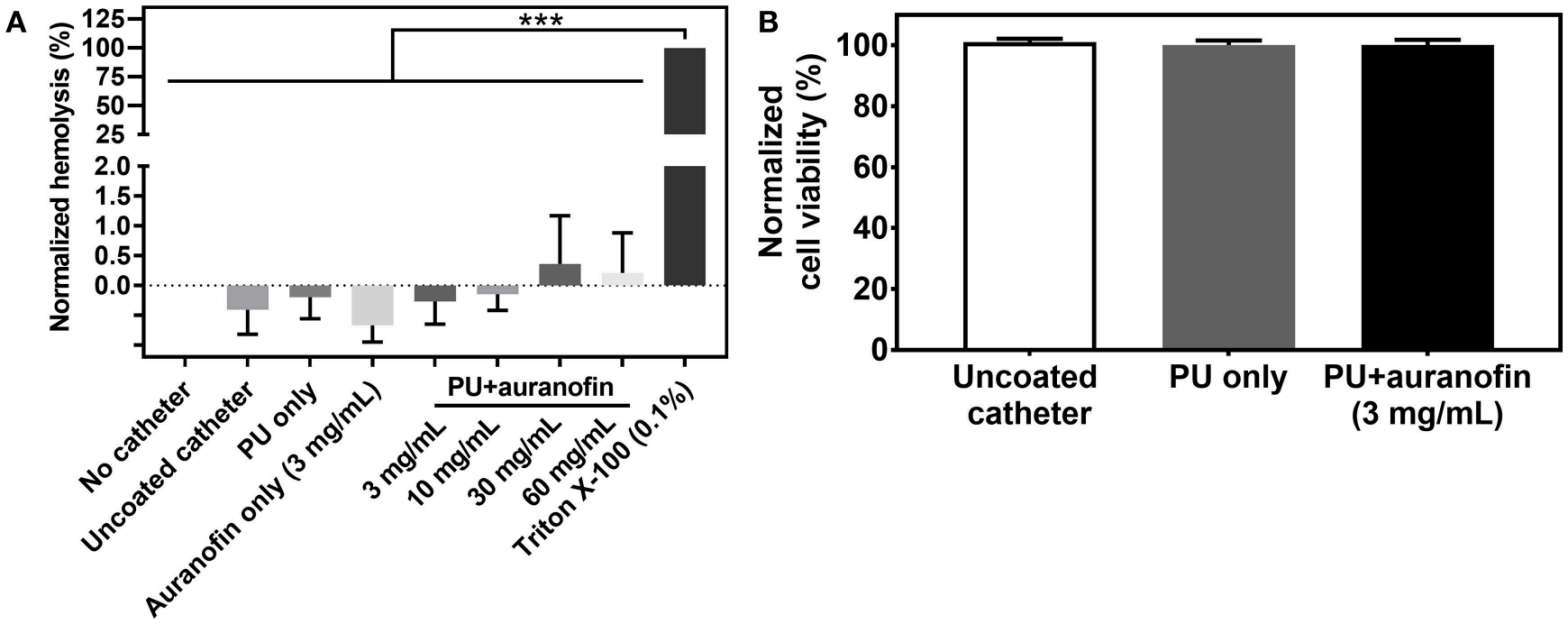
Cytotoxicity of PU+auranofin coatings. **(A)** Percent normalized hemolysis of hRBCs exposed to uncoated catheters, PU only, auranofin only (formulated at a 3 mg/mL auranofin coating concentration), PU+auranofin (formulated at 3, 10, 30, and 60 mg/mL auranofin coating concentrations) compared to negative controls of untreated hRBCs and a Triton X-100 incubated positive control. **(B)** Normalized HepG2 liver cell viability upon exposure to media incubated with uncoated catheters, PU only, and PU+auranofin (formulated at a 3 mg/mL auranofin coating concentration) catheters for 24 h. Data are shown as mean ± standard deviation. Statistical significance was evaluated using one-way ANOVA (*n* = 3) and is shown as ***p < 0.001 indicating statistical significance between the positive control (Triton X-100 with hRBCs) and other conditions tested. No statistical significance was noted between the other hemolysis conditions tested or between the different HepG2 viability conditions examined (*p* > 0.5).

The PU+auranofin coatings developed in this work are highly promising as antibacterial materials. Future translation of these materials requires that the materials are biocompatible. The drug coated catheters will eventually be employed as functioning devices; in this scenario, they will be exposed to circulating blood. For this reason, we tested the auranofin coated devices in the presence of human erythrocytes to see if the various drug coating concentrations could incite lysis. The hemolysis of PU+auranofin coatings at all formulations developed (i.e., 3, 10, 30, and 60 mg/mL of auranofin coating concentrations), PU only coatings, auranofin only coatings (formulated using a 3 mg/mL auranofin solution), and uncoated catheters was compared with hRBC negative and positive controls (i.e., untreated and Triton X-100 treated hRBCs, respectively), as shown in Figure 8A. We did not observe any significant difference in normalized hemolysis between any of the tested coating groups and the untreated controls, indicating excellent hemocompatibility of the PU+auranofin coated catheters.

We also evaluated the cytotoxicity of PU+auranofin coatings formulated using 3 mg/mL auranofin, PU only, and uncoated catheters incubated in cell culture media for 24 h on hepatocellular carcinoma cells. HepG2 cells were selected due to their widespread use as *in vitro* models for liver metabolism of toxins (Guillouzo et al., 2007). Initially we examined the viability of HepG2 cells with non-coating incorporated auranofin (Figure S2). We observed that the half maximal inhibitory concentration (IC_50_) for viability for non-coating incorporated auranofin fell between 16 and 32 μg/mL, which corresponds with IC_50_ values of auranofin previously reported for HepG2 cells (Liu et al., 2015). Figure 8B shows the percentage of viable cells upon exposure to catheter release media. None of the formulations tested affected the viability of HepG2 cells compared to a no catheter control. From the *in vitro* media release studies, a concentration of 3 μg/mL auranofin for the 24 h release sample could be estimated, falling well below the IC_50_ concentration. The catheter alone and PU alone coating was not expected to have any effect on the cells, as previously FDA approved materials. Similarly, auranofin is typically administrated orally to patients at an auranofin concentration of 6 mg/mL per day for antirheumatic therapy and has demonstrated no cumulative toxicity during long-term treatments (Egsmose et al., 1995). Therefore, auranofin has terrific potential to be utilized as an antibacterial and antibiofilm therapy without significant concern for human toxicity.

## Conclusions

Solvent casting PU+auranofin catheter coatings yielded materials that prevented the attachment of MRSA and the accumulation of bacteria that enables biofilm formation. Auranofin release profiles estimated in bacteria media demonstrated the potential to achieve 26 days of above MIC release for specific formulations of this coating. A large initial release in the first day was followed by a slow sustained release. MRSA growth inhibition was observed between 8 and 26 days depending on the auranofin concentration utilized during coating formation. These coatings exceed the maximal 2 week period of efficacy observed for previously reported antimicrobial catheters. Most importantly, the coatings were capable of completely preventing MRSA biofilm formation, a property unique to the combined PU+auranofin coating and not observed with auranofin or PU alone. The PU+auranofin coating did not adversely affect catheter structure. Finally, we observed that these coatings are non-toxic to healthy hRBCs and HepG2 cells, important for future preclinical and clinical translation of these products. Intravascular catheters can be used over a 72 to 96 h time period (Brown and Rowland, 2013); adding an inhibitory drug such as auranofin in the form of a sustained release coating can prevent infection by planktonic and biofilm bacteria, potentially limiting CRBSIs.

## Author Contributions

HL, SS, and NV-G contributed equally to this work. HL, SS, NV-G, and NT designed and carried out experiments. AS, EM, and BF designed experiments and directed research. HL, NV-G, SS, and AS wrote the manuscript. All authors analyzed results, revised the manuscript, and approved of the final version.

### Conflict of Interest Statement

The authors declare that the research was conducted in the absence of any commercial or financial relationships that could be construed as a potential conflict of interest.
