# Supplementary material for: Auranofin Releasing Antibacterial and Antibiofilm Polyurethane Intravascular Catheter Coatings
Source: Front Cell Infect Microbiol. 2019 Feb 28;9:37. doi: 10.3389/fcimb.2019.00037 (PMC6403144; doi:10.3389/fcimb.2019.00037)
Supplement: Supplementary file 1 [file Data_Sheet_1.pdf]

Supplementary Material

Auranofin Releasing Antibacterial and Antibiofilm Polyurethane Intravascular Catheter Coatings

Hanyang Liu,[1†] Shashank Shukla,[1†] Noel Vera-González,[1†] Nagendran Tharmalingam,[2] Eleftherios Mylonakis,[2] Beth Burgwyn Fuchs,[2] and Anita Shukla[1*]

[1]School of Engineering, Center for Biomedical Engineering, Institute for Molecular and Nanoscale Innovation, Brown University, 182 Hope Street Box D, Providence, RI, USA

[2]Division of Infectious Diseases, Rhode Island Hospital, Alpert Medical School and Brown University, 593 Eddy Street, Providence, RI, USA

[†]These authors have contributed equally to this work and are co-first authors.

*** Correspondence:**
Dr. Anita Shukla
anita_shukla@brown.edu

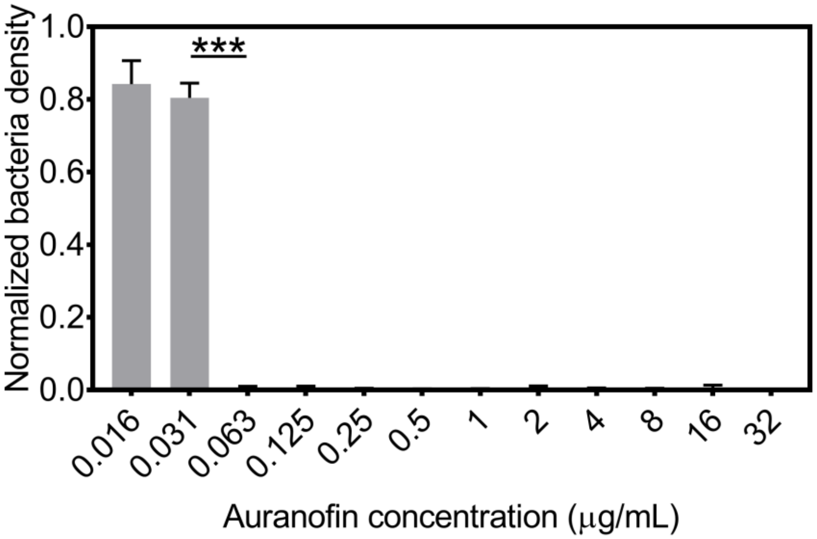

Figure S1: MIC of auranofin against MRSA USA 300. Data are shown as mean ± standard deviation where *** $p < 0.001$ between auranofin concentrations evaluated using one-way ANOVA (n = 3).

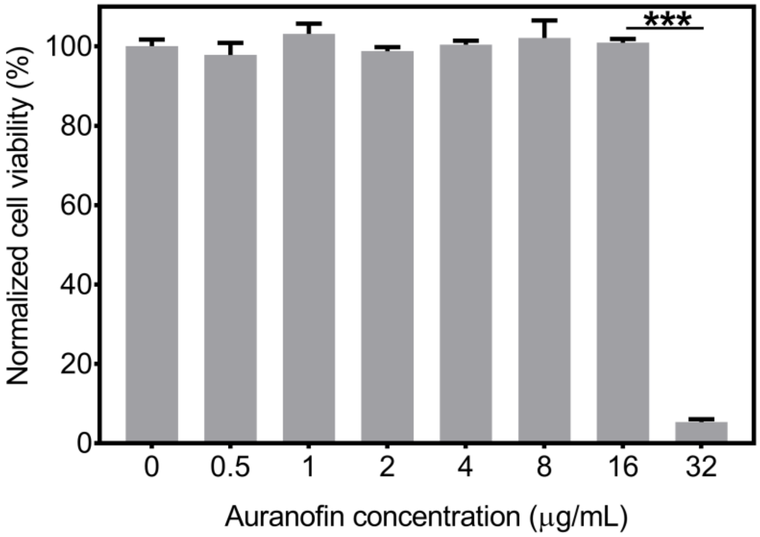

Figure S2: Auranofin toxicity to liver hepatocellular carcinoma (HepG2) cells. Data are shown as mean ± standard deviation where *** $p < 0.001$ between auranofin concentrations evaluated using one-way ANOVA (n = 3).
